# Mechanism of the crude extract from the whole herb of *Plantago asiatica L.* in alleviating lower limb ischemia-reperfusion injury

**DOI:** 10.1515/biol-2025-1381

**Published:** 2026-11-02

**Authors:** Jiangpeng Wu, Shenmei Wu, Jun Lv, Mengjiao Zhu, Jin Peng, Chuanzhu Li, Teng Gao, Guiyun Jin, Tang Deng

**Affiliations:** Department of Interventional Radiology and Vascular Surgery, Key Laboratory of Emergency and Trauma of Ministry of Education, The First Affiliated Hospital of Hainan Medical University, Hainan Medical University, Haikou 571199, China; Department of Radiology, Hainan Modern Women and Children’s Hospital, Haikou 570311, China

**Keywords:** lower limb ischemia-reperfusion injury, network pharmacology, apoptosis, oxidative stress, *Plantago asiatica L.*

## Abstract

This study evaluated the protective effects of *Plantago asiatica L.* (PAL) extract against acute lower limb ischemia-reperfusion injury (LL-IRI). Network pharmacology and molecular docking identified core targets associated with inflammation, oxidative stress, and apoptosis and indicated stable interactions betweenPAL constituents and these targets. *In vivo*, PAL improved skeletal muscle pathology and ultrastructural injury in LL-IRI rats, reduced interleukin-1β (IL-1β) and malondialdehyde (MDA) levels, restored superoxide dismutase (SOD) and catalase (CAT) activities and Bcl-2 expression, and suppressed Bax and cleaved Caspase-3. *In vitro*, PAL promoted the proliferation and migration of lipopolysaccharide (LPS)-injured C2C12 cells, stabilized mitochondrial membrane potential, and modulated apoptosis-related proteins. These findings support the view that PAL protects against LL-IRI by attenuating inflammation and oxidative stress and by regulating apoptosis.

## 1 Introduction

Lower limb ischemia-reperfusion injury (LL-IRI) is a serious complication that can occur after reperfusion therapy for occluded lower limb arteries [1]. Its clinical presentation is heterogeneous: in addition to skeletal muscle necrosis and local pain, patients may develop atypical manifestations such as edematous blisters and tissue necrosis. Disease progression can cause irreversible tissue injury, intense inflammatory activation, renal failure, and even multiple organ dysfunction syndrome [2]. Apoptosis is a major form of cell death that aggravates tissue damage within the complex pathological setting of LL-IRI [3]. This gene-regulated process involves coordinated biochemical reactions and structural changes in cells. In ischemia-reperfusion models, gastrocnemius fibers become loosely arranged, the sarcolemma and striations are disrupted, interstitial swelling is prominent, and areas of myofiber breakdown and necrosis appear together with increased apoptotic cells [3]. During reperfusion, inflammation and oxidative stress can activate mitochondria-dependent apoptotic pathways. In a mouse tourniquet model of hindlimb ischemia-reperfusion, reperfusion increased mitochondrial superoxide production, opened the mitochondrial permeability transition pore (mPTP), and induced apoptosis in gastrocnemius muscle cells. Tempol or coenzyme Q10 reduced inflammatory infiltration and apoptosis, supporting the involvement of mitochondrial apoptotic signaling [4]. Ischemia-reperfusion injury (IRI) has attracted broad research attention, but LL-IRI remains relatively understudied [5]. Controlled reperfusion (CR) can reduce the composite 4-week risk of amputation or death in acute limb ischemia (ALI) from 33 to 8 % [6], and continuous renal replacement therapy (CRRT) can improve myonephropathic metabolic syndrome by lowering blood myoglobin and creatine kinase (CK) while maintaining homeostasis [7]. However, these invasive approaches are costly and carry a risk of complications, limiting their use in many patients. Effective, affordable, noninvasive, and easily applicable interventions are therefore needed to improve the prognosis of LL-IRI.

*Plantago asiatica L.* is widely cultivated in Hainan and has a long history of medicinal use. In this study, *P. asiatica L.* extract is abbreviated as PAL. The plant is recorded in the Shennong Ben Cao Jing (Classic of the Materia Medica), and the whole herb has been used for anti-inflammatory, antioxidant, and other therapeutic purposes [8]. PAL contains several active constituents, including aucubin (AU), plantagoside, homoplantaginin, and β-sitosterol [9], 10]. Previous studies suggest that PAL may improve pulmonary artery remodeling by reducing hypoxia-inducible factor-1α (HIF-1α) expression in lung tissue from rats with hypoxic pulmonary hypertension (HPH) and by suppressing inflammatory mediators such as intercellular adhesion molecule-1 (ICAM-1) and transforming growth factor-β (TGF-β) [11], [12], [13], [14]. Hydroalcoholic extracts of Plantago have also been reported to reduce renal apoptosis and improve kidney function in doxorubicin-induced nephropathy [15], and combined treatment with vitamin E may counteract cisplatin-induced nephrotoxicity [16]. These findings provide a rationale for examining the potential effects of PAL in LL-IRI. Traditional Chinese medicine (TCM), with its multi-target profile, low toxicity, affordability, and noninvasive administration, is a promising source of candidate therapies for LL-IRI. Contemporary pharmacological studies further support the anti-inflammatory and antioxidant properties of PAL. Among its constituents, AU can scavenge free radicals [17], suppress inflammatory responses, and inhibit apoptosis [18], [19], [20].

In 2007, Hopkins introduced network pharmacology, a systems-biology and polypharmacology-based approach for identifying drug targets and mechanisms through drug-target-disease networks, multi-omics data, biological network structure, and signaling pathway analysis [21]. Its central concept is the network target, which emphasizes coordinated modulation of multiple signaling pathways and addresses the limitations of conventional single-target research. This framework is particularly suitable for TCM and for diseases driven by interacting mechanisms. For example, Li et al. [22] used network pharmacology to identify shared targets between Polygonati Rhizoma (Huangjing) and sarcopenia, highlighted the PI3K/Akt/mTOR pathway, and experimentally showed that polysaccharides improved mitochondrial function through this pathway. Shi et al. [23] integrated multiple data sources to predict the targets of Jiegeng Gancao Decoction in ulcerative colitis and used proteomics and molecular docking to clarify its anti-inflammatory and healing mechanisms. Network pharmacology can therefore move efficiently from target prediction to mechanistic validation. Because PAL contains multiple active components and LL-IRI involves multi-target pathological interactions, this approach is well suited for identifying the principal active components of PAL, defining key LL-IRI-related targets and pathways, and constructing a PAL-component-target-disease network to guide experimental validation.

This study investigates the therapeutic targets and molecular pathways involved in the effects of PAL on LL-IRI. By integrating network pharmacology with *in vivo* and *in vitro* experiments, it provides mechanistic evidence and a potential basis for developing natural herbal interventions for LL-IRI.

## 2 Materials and methods

### 2.1 Network pharmacology

#### 2.1.1 Collection of active components and targets of PAL

The chemical constituents of PAL were collected from the Traditional Chinese Medicine Systems Pharmacology Database and Analysis Platform (TCMSP), Bioinformatics Analysis Tool for Molecular Mechanism of Traditional Chinese Medicine (BATMAN-TCM), Encyclopedia of Traditional Chinese Medicine (ETCM), and HERB databases, and the active components were screened. The TCMSP screening criteria were oral bioavailability (OB) ≥ 30 % and drug-likeness (DL) ≥ 0.18. Target gene names were standardized using the official UniProt ID mapping tool, after which duplicate targets were removed.

#### 2.1.2 Screening of PAL and disease-related targets

Disease-related targets were retrieved from the GeneCards (score > 20) and Online Mendelian Inheritance in Man (OMIM) databases using “acute lower limb ischemia-reperfusion injury” as the search term. These targets were intersected with the compound-related targets identified in Section 2.1 to obtain candidate targets through which PAL may act on LL-IRI.

#### 2.1.3 Construction and analysis of the drug-compound-target-disease network

Interactions between PAL active components and their targets were imported into Cytoscape 3.9.1 to construct a network. Network topology was then analyzed, and node degree values were calculated.

#### 2.1.4 Construction and analysis of the protein-protein interaction (PPI) network

The PPI network was constructed and imported into Cytoscape for core-target screening.

#### 2.1.5 Gene ontology (GO) functional enrichment and Kyoto encyclopedia of genes and genomes (KEGG) pathway enrichment analyses

GO functional enrichment and KEGG pathway enrichment analyses were performed for the PAL- and disease-related targets. KEGG results were visualized as bubble plots.

#### 2.1.6 Construction and analysis of the drug-compound-target-pathway-disease network

Key pathways, together with their principal targets and associated active components, were selected to construct the network.

#### 2.1.7 Molecular docking

The three-dimensional structures of the key PAL active components and their corresponding target proteins were obtained for molecular docking analysis, and the results were visualized.

### 2.2 Preparation of the Plantago decoction

After the whole herb of PAL was dried in the sun, 500 g of material was soaked in warm water for 30 min and decocted twice. The two decoctions were combined, filtered three times until clear, and concentrated to 100 mL to obtain a 5 g/mL stock solution. The solution was then passed through a 0.22 μm filter membrane. Before administration, the stock solution was diluted fivefold with sterile distilled water to obtain a 1 g/mL working suspension.

### 2.3 Cell culture

C2C12 mouse embryonic myoblasts, obtained from Shanghai Baidi Biotechnology, were cultured in high-glucose Dulbecco’s modified Eagle medium (DMEM) supplemented with 10 % fetal bovine serum (FBS) and 1 % penicillin-streptomycin. Cells were passaged and cryopreserved in liquid nitrogen according to standard procedures. Cells below passage 10 were maintained at 37 °C in an atmosphere containing 5 % CO_2_ and 20 % O_2_.

### 2.4 Animal models and experimental grouping

#### 2.4.1 Animals and ethics

Eighteen male Sprague-Dawley (SD) rats, aged 6–8 weeks and weighing approximately 160 g (±10 g), were obtained from Hunan Slack Kingda Laboratory Animal Co., Ltd. The animals were housed at the Animal Experiment Center of Hainan Medical University at 22 °C (±1 °C) and 55 % humidity.

**Ethical approval:** The research related to animal use has been complied with all the relevant national regulations and institutional policies for the care and use of animals, and has been approved by the Ethics Committee of Hainan Medical College (approval numer: HYLL-2024-124).

#### 2.4.2 *In vivo* experimental design

Rats were randomly assigned to three groups. The control group (CON) received 1 mL of saline daily for 4 days and underwent no additional intervention. The LL-IRI group received 1 mL of saline daily for 4 days; on Day 4, both groin roots were constricted with an orthodontic rubber band (3.5 oz, 1/46.35 mm) for 3 h to induce bilateral lower limb ischemia. The band was then removed, and the rats were euthanized 2 h after reperfusion. The PAL pretreatment group received PAL (1 g/mL) by gavage at 5 g/kg for three consecutive days, followed by LL-IRI modeling on Day 4. The entire experiment was conducted in an SPF-grade animal facility maintained at 22 ± 1 °C. Immediately after surgery, rats were placed on a heating pad maintained at 37 °C to preserve body temperature and reduce the impact of hypothermia on IRI outcomes.

#### 2.4.3 Tissue collection

Under general anesthesia induced by intraperitoneal injection of 3 % sodium pentobarbital (50 mg/kg), the rats were euthanized by cervical dislocation. Skeletal muscle samples from both hindlimbs were collected for hematoxylin-eosin (HE) staining, transmission electron microscopy (TEM), terminal deoxynucleotidyl transferase dUTP nick-end labeling (TUNEL) staining, multiplex tissue immunofluorescence, and Western blot analysis.

### 2.5 *In vitro* experimental design

Untreated C2C12 cells served as the control group. To establish the injury model, cells were exposed to 100 μg/mL lipopolysaccharide (LPS) for 48 h. For PAL treatment, cells were pretreated with PAL at 5 μg/mL (low-dose group, L) or 10 μg/mL (high-dose group, H) for 24 h, after which LPS was added to induce injury.

### 2.6 HE staining of gastrocnemius muscle

The lateral gastrocnemius muscle from SD rats was fixed in 4 % formaldehyde, dehydrated, embedded in paraffin, sectioned, dewaxed, rehydrated, and stained with HE.

### 2.7 Transmission electron microscopy of cellular ultrastructure

Specimens were fixed in 2.5 % glutaraldehyde for 12 h, washed with phosphate-buffered saline (PBS), and postfixed with osmium tetroxide. After dehydration through a graded ethanol series, the samples were treated with propylene oxide for 30 min and infiltrated with a 1:1 mixture of propylene oxide and epoxy resin for 2 h. The samples were then embedded, polymerized, trimmed, and sectioned. Sections were stained with uranyl acetate in the dark, counterstained with lead citrate, and dried overnight at room temperature before examination and imaging by TEM.

### 2.8 Multiplex tissue immunofluorescence staining

Paraffin sections were deparaffinized and rehydrated, followed by antigen retrieval and blocking with 5 % bovine serum albumin (BSA) for 30 min at room temperature. A mixture of primary antibodies against the target antigens was applied and incubated overnight at 4 °C. After three 5-min washes with phosphate-buffered saline with Tween-20 (PBST), fluorophore-conjugated secondary antibodies were added and incubated for 1 h in the dark at room temperature. Nuclei were counterstained with 4′,6-diamidino-2-phenylindole (DAPI; 1 μg/mL) for 5 min, and the sections were mounted with antifade medium. Images were acquired using a confocal microscope, and colocalization and expression levels were analyzed with ImageJ.

### 2.9 Antibody sources and concentrations

All antibodies were purchased from Servicebio (Wuhan, China): Bax (1:400), Bcl-2 (1:500), and Caspase-3 (Cas-3; 1:500).

### 2.10 TUNEL staining

Paraffin sections were deparaffinized and rehydrated, followed by proteinase K digestion, permeabilization, and equilibration at room temperature. TUNEL reaction solution (Servicebio, Wuhan, China) was then added, and the sections were incubated at 37 °C for 1 h. Nuclei were counterstained with DAPI, after which the sections were mounted and examined under a fluorescence microscope. Fluorescence intensity was quantified using ImageJ.

### 2.11 Half-maximal inhibitory concentration (IC50) determination

Cells were divided into nine groups: one complete-medium control group and eight LPS-treated groups exposed to 0.1, 0.5, 1, 50, 100, 200, 300, or 500 μg/mL LPS. Cells were seeded in 96-well plates and allowed to adhere before exposure to the corresponding LPS concentrations. After incubation at 37 °C for 48 h, Cell Counting Kit-8 (CCK-8) reagent was added according to the manufacturer’s instructions, followed by incubation for 3–4 h. Absorbance was measured at 450 nm, and the half-maximal inhibitory concentration (IC50) curve was generated.

### 2.12 Western blot analysis

Protein samples from rat hindlimb skeletal muscle and C2C12 cells were subjected to electrophoresis, membrane transfer, blocking, and antibody incubation. The membranes were then washed to remove nonspecifically bound antibodies, and protein bands were visualized using a chemiluminescence imaging system. The antibodies were purchased from Proteintech (Wuhan, China): Bcl-2 (1:1,500), Caspase-3 (Cas-3; 1:2,000), and Bax (1:8,000).

### 2.13 CCK-8 assay for cell proliferation

Cells were divided into nine groups: a complete-medium control group and PAL-treated groups exposed to 4, 6, 8, 10, 25, 50, 100, or 200 μg/mL PAL. Cells were seeded in 96-well plates and, after attachment, treated with 100 µL of PAL solutions prepared in complete medium containing 5 % FBS. One plate was used to assess the effect of PAL alone on cell viability. In the second plate, the medium was removed after cell attachment and replaced with 100 µL of 100 μg/mL LPS prepared in complete medium containing 3 % FBS. Cells were cultured at 37 °C for 48 h and then processed using the same method as the first plate.

### 2.14 Biochemical assays for IL-1β, MDA, SOD, and CAT

An IL-1β assay kit from Qingdao Baizhou and malondialdehyde (MDA), superoxide dismutase (SOD), and catalase (CAT) kits from Nanjing Jiancheng were used to assess inflammatory and oxidative stress markers in C2C12 cell lysates, cell culture supernatants, and rat serum. After injury modeling, the culture supernatant from each well was transferred to sterile centrifuge tubes and centrifuged at 1,000×*g* for 5 min at 4 °C to remove suspended cells and debris. The resulting supernatant was collected as the supernatant sample. Adherent cells were gently rinsed twice with cold PBS, lysed on ice for 30 min with an appropriate volume of cell lysis buffer, and centrifuged at 12,000×*g* for 15 min at 4 °C. The supernatant was collected as the lysate sample. IL-1β, MDA, SOD, and CAT levels or activities were measured according to the instructions for the corresponding enzyme-linked immunosorbent assay (ELISA) or biochemical kits. Standards and diluted samples were added to a precoated 96-well plate and incubated at 37 °C, followed by washing and sequential addition of biotinylated detection antibody and horseradish peroxidase (HRP)-conjugated streptavidin. After color development with 3,3′,5,5′-tetramethylbenzidine (TMB) substrate and reaction termination, absorbance was measured at 450 nm, and marker concentrations were calculated from the standard curve.

### 2.15 Scratch assay for cell migration

Cells were seeded into four 6-well plates according to group assignment and monitored after 24 h in a 37 °C incubator. When cells reached approximately 90 % confluence, a straight scratch was made with a 200 µL pipette tip. The corresponding treatments were then added, and the plates were returned to the incubator for 72 h. Microscopic images were captured every 24 h to record scratch closure in each group. The scratch closure rate was calculated using ImageJ.

### 2.16 Statistical analysis

Results are expressed as the mean ± standard deviation (*x* ± SD). Group means were compared using one-way analysis of variance (ANOVA) followed by *t*-tests where appropriate. *P* < 0.05 was considered statistically significant. All data were normally distributed and were analyzed using GraphPad Prism software.

## 3 Results

### 3.1 Network pharmacology reveals a multi-target mechanism for PAL in LL-IRI

Using a network pharmacology framework, this study examined the potential targets and signaling pathways through which PAL may act in LL-IRI. The Venn diagram (Figure 1A) identified 145 shared targets between PAL and LL-IRI. A component-target interaction network was then constructed (Figure 1B), and PPI analysis (Figure 1C) highlighted key PAL-related targets in LL-IRI, including TNF, IL-6, IL-1β, Bcl-2, Bax, and Caspase-3. GO enrichment analysis (Figure 1D), covering biological process (BP), molecular function (MF), and cellular component (CC) categories, indicated that PAL-related targets were concentrated in biological processes associated with inflammation, oxidative stress, and apoptosis. KEGG enrichment analysis (Figure 1E) further suggested that PAL may protect against LL-IRI by modulating apoptosis, VEGF, PI3K-Akt, MAPK, and related signaling pathways, consistent with a multi-pathway regulatory mechanism.

**Figure 1:**
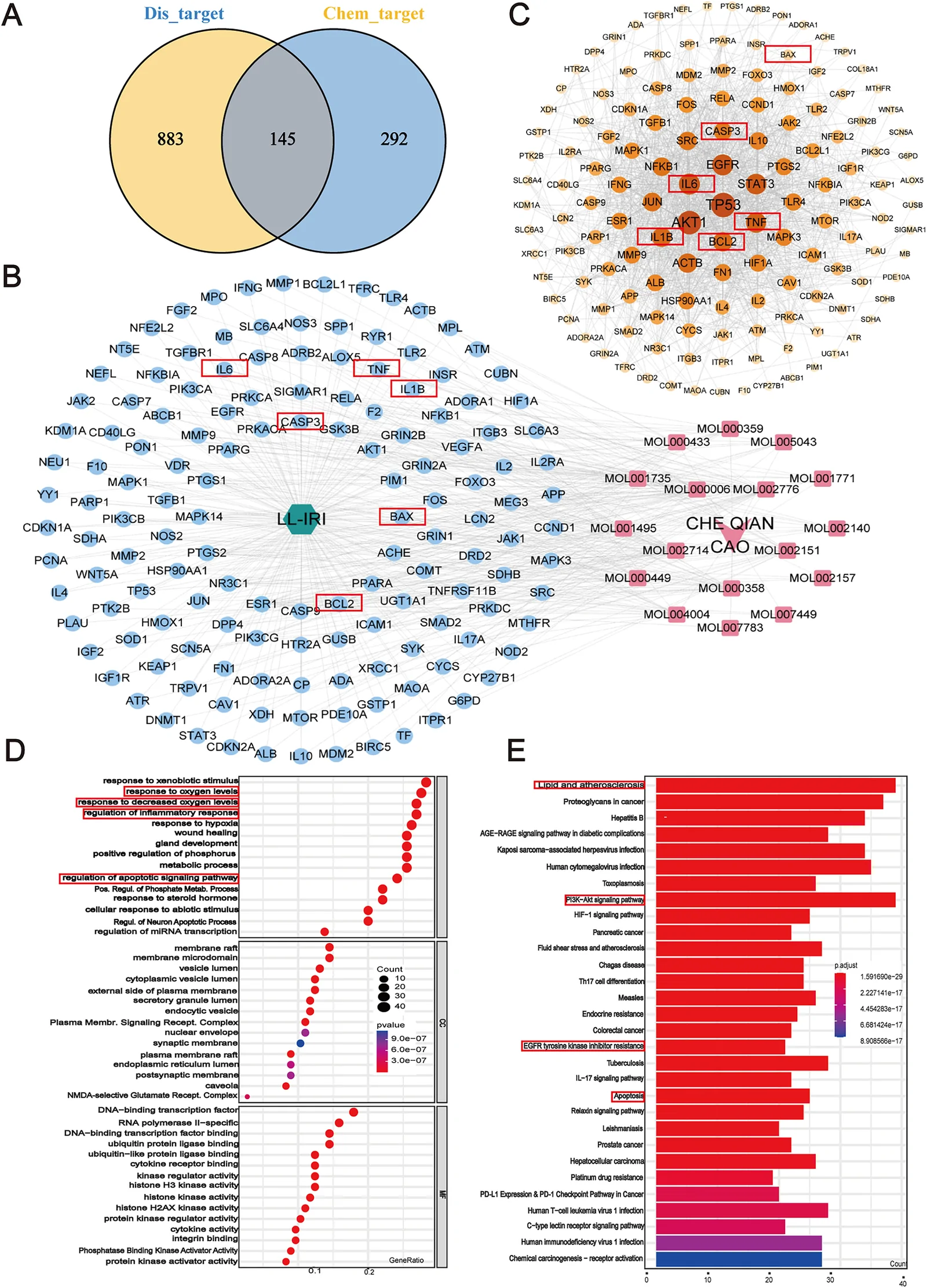
Network pharmacology analysis of interaction targets and pathways between PAL and LL-IRI (A) Venn diagram showing the overlap between disease-related targets and PAL targets. GeneCards (score > 20) and OMIM yielded 1,028 disease-related targets; intersection with the 292 targets associated with PAL active components identified 145 shared targets. (B) Network showing interactions among the drug, active components, targets, and disease. The network contains 164 nodes (17 active components and 145 shared targets) and 452 interactions, highlighting the multi-component and multi-target characteristics of PAL. (C) PPI network of target proteins. Node size and color reflect degree values and show the top 30 core targets and their interactions. (D) Bar plot of GO enrichment analysis showing the 15 most significantly enriched categories across BP, MF, and CC terms (*P* < 0.05). The *x*-axis indicates the number of enriched targets. (E) Bubble plot of KEGG enrichment showing the 30 most enriched pathways (*P* < 0.05). Bubble size indicates the number of targets, and color indicates the adjusted *P* value.

### 3.2 Network construction analysis and molecular docking

On the basis of the target analysis, a drug-compound-target-pathway-disease interaction network was constructed (Figure 2A). Network topology showed that one active component could act on multiple targets and that one target could be regulated by several components, reflecting the multi-component and multi-target features of PAL. Pathway connections through shared targets also suggested that PAL may influence LL-IRI through coordinated regulation of several signaling pathways. To assess the binding potential of active components with key targets, six major components with high degree values in the network (MOL000449, MOL000433, MOL000358, MOL004004, MOL002714, and MOL000006) were selected for molecular docking with the top 10 core targets from the PPI network, including AKT1, Bcl-2, IL-1β, IL-6, TNF, and p53 (Figure 2B–H). All tested component-target pairs showed binding free energies below −5.0 kcal/mol, and several were below −7.5 kcal/mol, indicating favorable binding. These results provide structural support for interactions between PAL components and LL-IRI-related targets and further support the multi-target activity of PAL.

**Figure 2:**
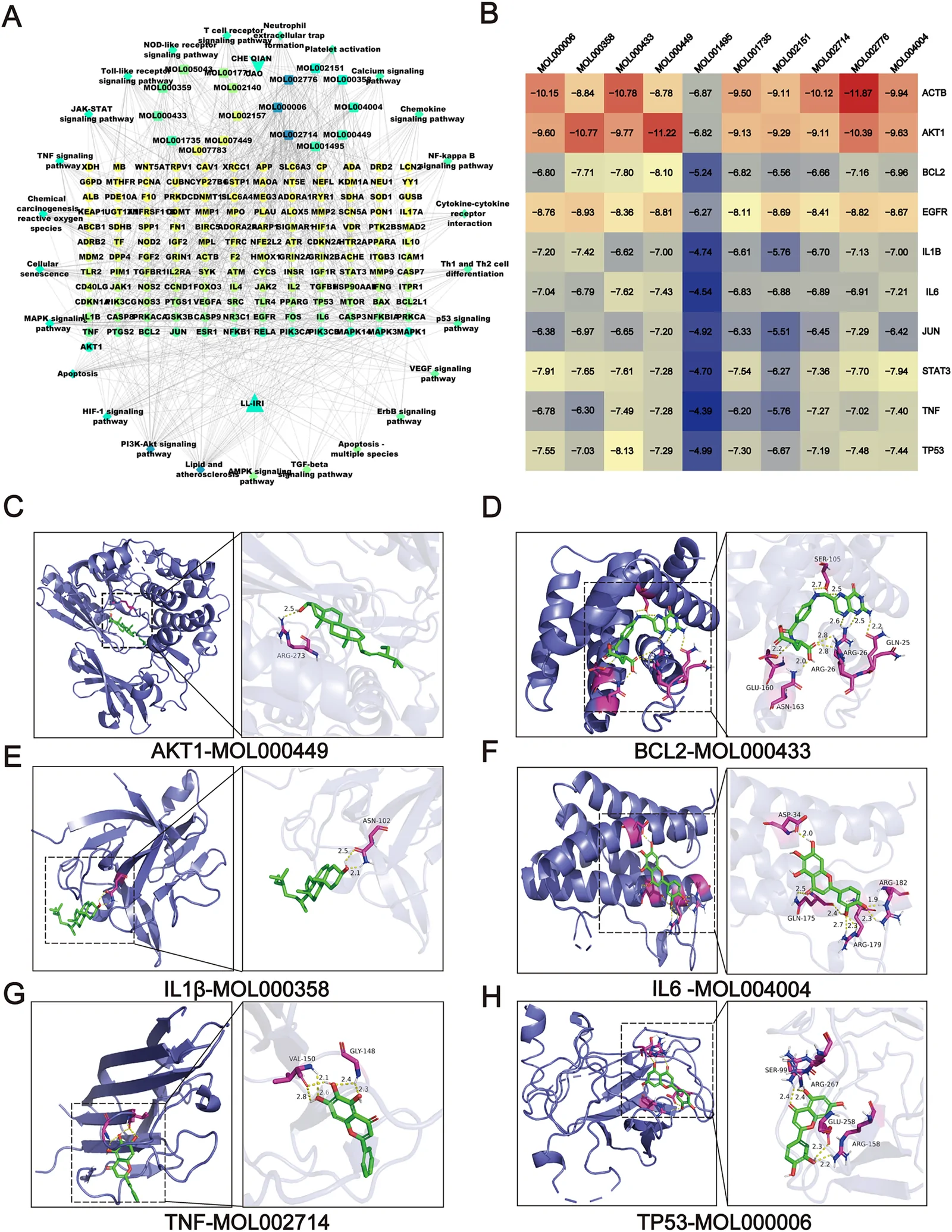
Construction of the drug-compound-target-pathway-disease network and molecular docking (A) multilayer network showing the relationships among PAL active components, their targets, associated signaling pathways, and LL-IRI. The network includes 189 nodes and 795 edges, comprising 17 active components, 145 targets, 25 pathways, and disease-related information. Different shapes represent different element types, and color intensity reflects node degree. (B) Heatmap of molecular docking binding energies. The color scale ranges from −11.3 to +10.1 kcal/mol. Binding energies of ≤−5.0 kcal/mol indicate stable interactions, whereas values of ≤−7.5 kcal/mol indicate stronger compatibility and higher affinity. (C–H) Molecular docking models showing interactions between PAL active components and AKT1, Bcl-2, IL-1β, IL-6, TNF, and TP53. Protein receptors are shown in purple, active components in green, and key interaction sites, including hydrogen bonds, in pink.

### 3.3 PAL alleviates LL-IRI-induced skeletal muscle tissue injury

To assess the protective effects of PAL against LL-IRI, the treatment conditions were selected on the basis of previous work by our group and other studies. PAL was administered by gavage at 5 g/kg using a 1 g/mL working suspension [11], [12], [13], [14]. Outcomes were evaluated at three levels: gross morphology, histopathology, and ultrastructure. Rats in the LL-IRI model group showed pronounced lower-limb swelling and increased skin tension (Figure 3A); after PAL treatment, these pathological changes were markedly improved, with reduced swelling and lower skin tension. Hematoxylin-eosin (HE) staining (Figure 3B) showed disorganized muscle fibers, enlarged intercellular spaces, and marked inflammatory cell infiltration in the model group. By contrast, the PAL-treated group showed more intact muscle fibers and reduced inflammatory infiltration. Transmission electron microscopy (TEM) (Figure 3C) further showed that PAL attenuated ultrastructural injury, including mitochondrial swelling and myofibrillar disruption. Together, the gross morphological, histological, and ultrastructural data indicate that PAL reduces LL-IRI-induced skeletal muscle edema and structural injury.

**Figure 3:**
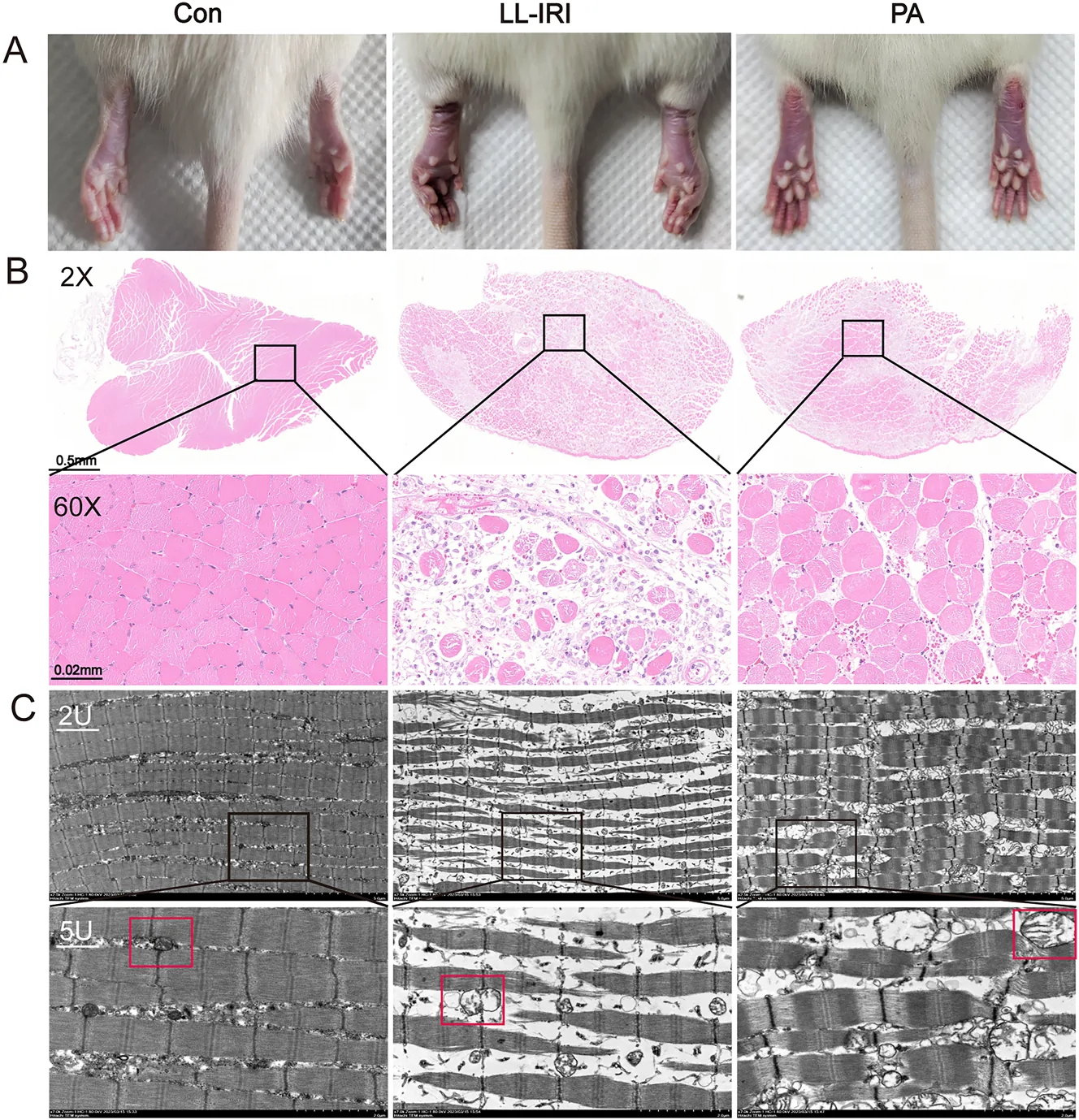
PAL alleviates LL-IRI-induced skeletal muscle tissue injury and edema (A) representative lower-limb images from each group. The LL-IRI model group showed marked swelling and increased skin tension, whereas PAL treatment reduced swelling and produced an appearance closer to normal. (B) Microscopic images of H&E-stained skeletal muscle tissue (scale bars: 0.5 mm at 2× and 0.02 mm at 60× magnification; mitochondria are enclosed by the red box). Muscle fibers were disorganized, intercellular spaces were enlarged, and inflammatory cells were abundant in the model group. The PAL-treated group showed better-preserved muscle fibers and fewer inflammatory cells. (C) TEM images of tissue ultrastructure (scale bars: 2 and 5 μm). In the model group, mitochondria were swollen and irregular, with indistinct cristae, and myofibrils were loosely arranged. PAL treatment produced more uniform mitochondrial morphology, clearer cristae, and more orderly myofilament arrangement. *n* ≥ 3. The red box indicates mitochondria.

### 3.4 PAL modulates apoptosis-related proteins and reduces LL-IRI-induced skeletal muscle cell death

To examine the effect of PAL on skeletal muscle cell apoptosis after LL-IRI, we assessed the expression and distribution of Bax, Bcl-2, and cleaved Caspase-3 by multiplex immunofluorescence staining (Figure 4A–D). Bax and Caspase-3 were mainly localized in the cytoplasm and were significantly increased in the LL-IRI model group (**P < 0.01), indicating activation of mitochondria-mediated intrinsic apoptosis. Bcl-2 was also predominantly cytoplasmic, but its expression was significantly reduced in the model group (**P < 0.01), indicating weakened anti-apoptotic capacity. PAL treatment reduced Bax and cleaved Caspase-3 levels and increased Bcl-2 expression (**P < 0.01), suggesting restoration of the balance among apoptosis-related proteins and inhibition of excessive apoptotic signaling. TUNEL staining supported these findings (Figure 4E and F): apoptotic cells increased in the LL-IRI model group and decreased significantly after PAL treatment. Overall, PAL alleviates LL-IRI-induced apoptosis in skeletal muscle cells by downregulating Bax and cleaved Caspase-3 and upregulating Bcl-2.

**Figure 4:**
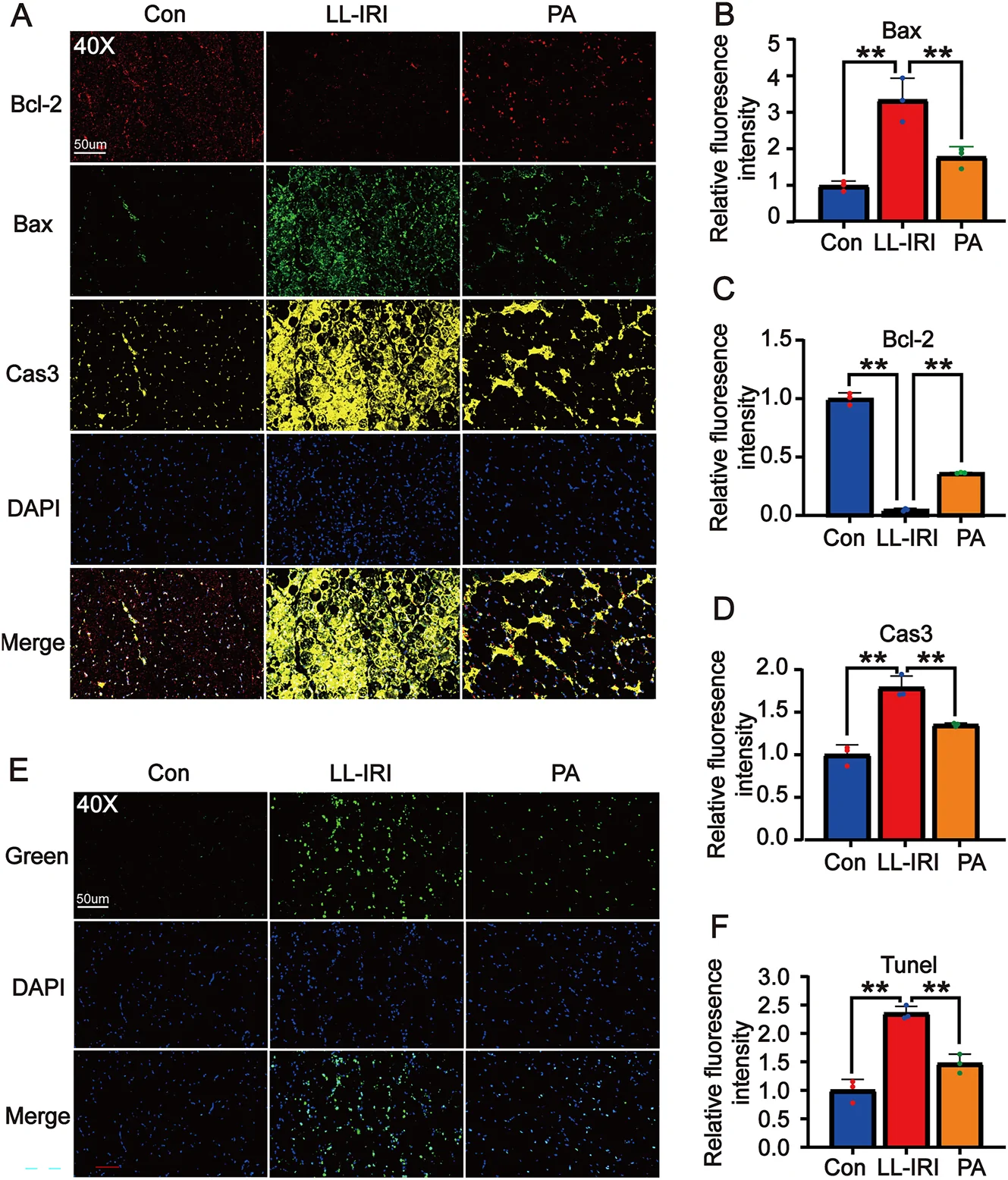
PAL alleviates LL-IRI-induced skeletal muscle apoptosis (A–D) immunofluorescence staining and quantitative analysis of Bax, Bcl-2, and Caspase-3 in skeletal muscle from rats in each group (scale bar: 50 μm). (E–F) TUNEL staining for apoptosis in skeletal muscle cells. Apoptotic cells are shown in green, and DAPI-stained nuclei are shown in blue (scale bar: 50 μm). *n* ≥ 3; *P < 0.05, **P < 0.01. PAL, LL-IRI + PAL.

### 3.5 Western blot validation of PAL-mediated regulation of the apoptotic pathway in LL-IRI

Western blot analysis was used to further evaluate the effects of PAL on apoptosis-related proteins after LL-IRI. Compared with the control group, the LL-IRI group showed significantly increased Bax and cleaved Caspase-3 (**P < 0.01) and reduced Bcl-2 (**P < 0.01), indicating activation of mitochondria-mediated apoptosis (Figure 5A–D). PAL treatment decreased Bax and cleaved Caspase-3 expression and increased Bcl-2 expression (**P < 0.01), confirming that PAL suppresses excessive activation of the apoptotic pathway.

**Figure 5:**
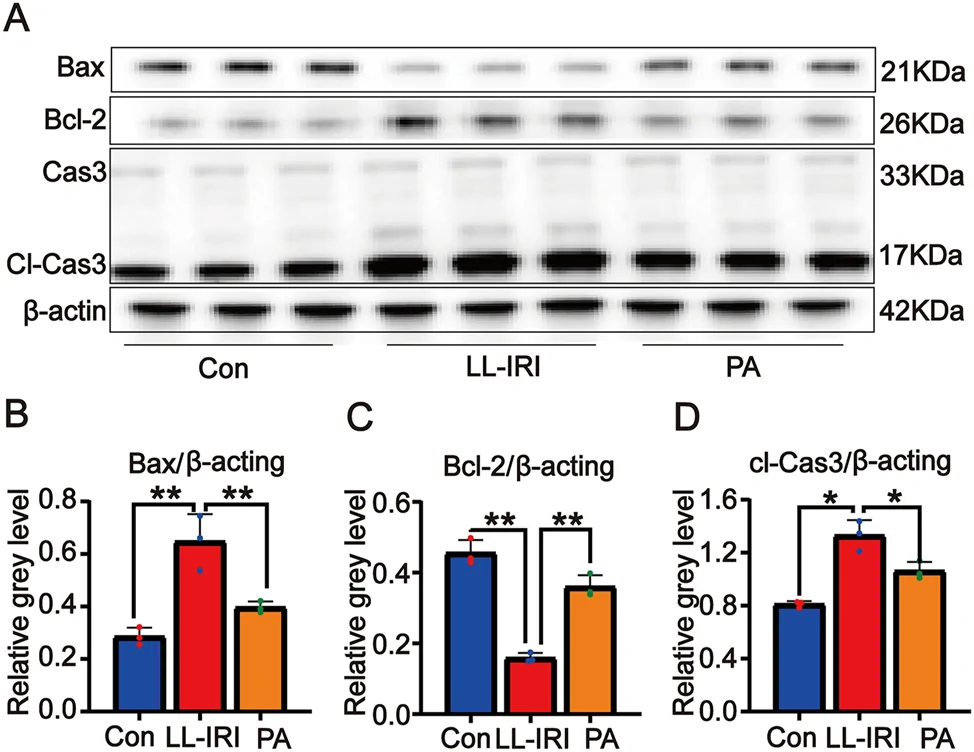
Regulatory effect of PAL on the apoptotic pathway (A–D) western blot analysis and quantification of Bax, Bcl-2, and cleaved Caspase-3 expression in each group. *n* ≥ 3; *P < 0.05, **P < 0.01. PAL, LL-IRI + PAL.

### 3.6 PAL promotes proliferation and migration after LPS stimulation and improves the oxidative and inflammatory microenvironment

To evaluate the regulatory effects of PAL on cellular function, IC50 and CCK-8 assays were used to examine the effects of different LPS and PAL concentrations on cell viability. The IC50 assay showed that 100 μg/mL LPS for 48 h reduced C2C12 cell viability by approximately 50 % (Figure 6A); this condition was therefore selected to establish the *in vitro* injury model. In untreated cells exposed to different PAL concentrations, CCK-8 results showed little change in viability between 4 and 25 μg/mL, whereas 50–200 μg/mL reduced viability, indicating that excessive PAL concentrations may impair cell growth. In LPS-injured cells pretreated with PAL, proliferation increased gradually between 4 and 10 μg/mL and remained relatively high at 10–25 μg/mL, whereas 50 μg/mL reduced viability. These results indicate a concentration-dependent effect of PAL, with beneficial effects within a limited range and inhibitory effects at higher concentrations.

**Figure 6:**
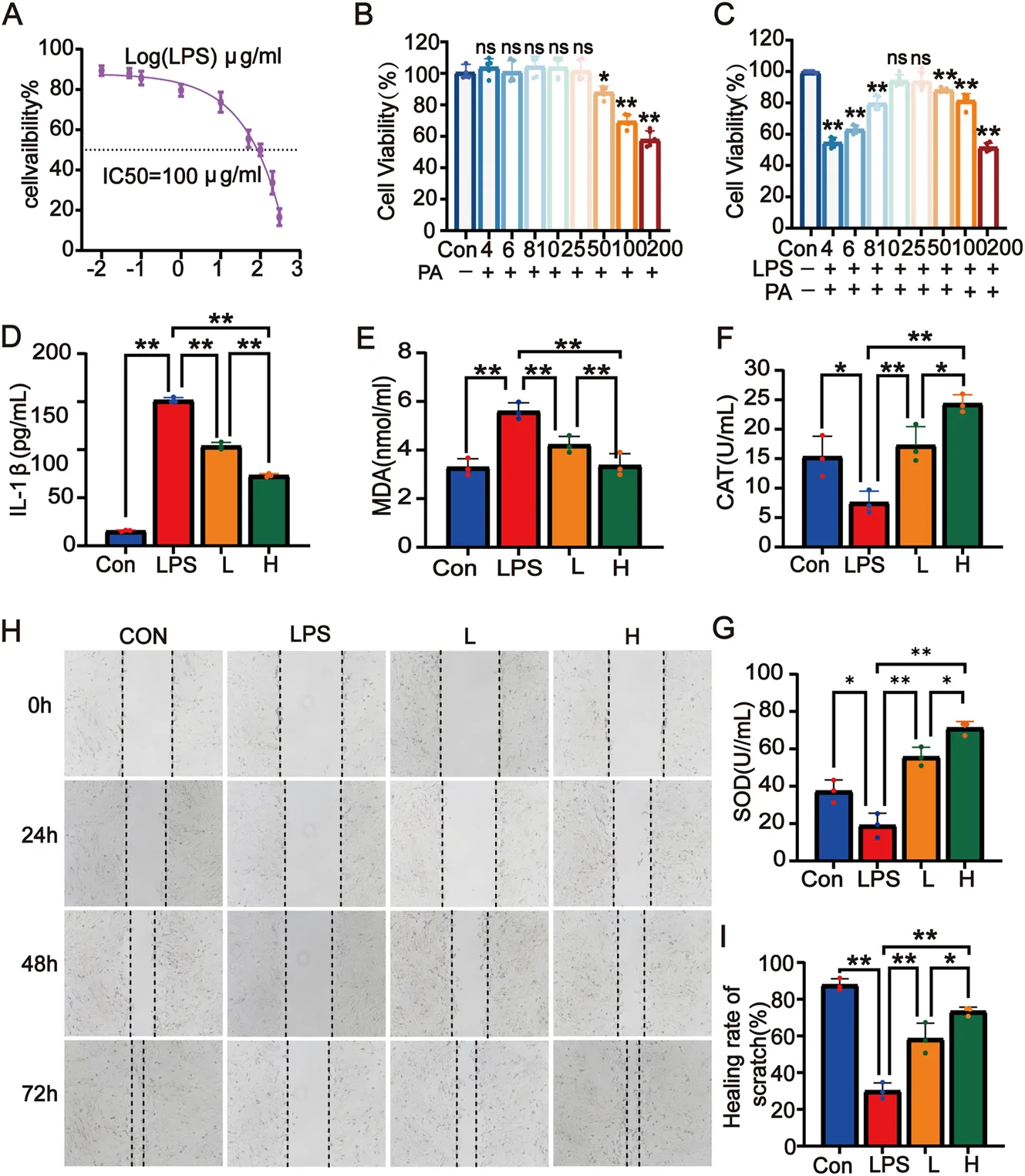
Effects of PAL on cell proliferation and migration after LPS stimulation (A) untreated C2C12 cells served as the control group. *In vitro* cytotoxicity was assessed after exposure to LPS concentrations of 0.01, 0.05, 0.1, 1, 10, 50, 100, 200, or 300 μg/mL for 48 h. (B) CCK-8 assay evaluating the effects of different PAL concentrations on C2C12 proliferation. (C) CCK-8 assay evaluating the effects of different PAL concentrations on cell viability after treatment with 100 μg/mL LPS. (H–I) representative scratch-assay images at 0, 24, 48, and 72 h and quantitative analysis of cell migration. *n* ≥ 3; *P < 0.05, **P < 0.01. L, LPS + 5 μg/mL PAL; H, LPS + 10 μg/mL PAL.

CCK-8 results indicated that PAL concentrations of 2–25 μg/mL did not reduce cell viability and were therefore considered noncytotoxic (Figure 6B). Additional experiments confirmed a concentration-dependent effect within this range (Figure 6C). Accordingly, 5 and 10 μg/mL were selected as the low-dose (L) and high-dose (H) concentrations for subsequent *in vitro* experiments. ELISA and biochemical assays were then used to assess oxidative stress and inflammation, and a scratch assay was used to evaluate cell migration. In the LPS group, IL-1β and MDA levels were significantly higher than in the control group (**P < 0.01), whereas SOD and CAT activities were reduced (*P < 0.05, **P < 0.01; Figure 6D–G). PAL treatment reduced IL-1β and MDA levels and restored SOD and CAT activities, with the high-dose group generally showing stronger effects than the low-dose group. The scratch assay (Figure 6H and I) showed that wound closure was significantly slower in the LPS model group than in the control group (**P < 0.01). PAL treatment increased wound closure, with a greater effect in the high-dose group (**P < 0.01). These findings indicate that PAL protects LPS-injured cells in a concentration-dependent manner by reducing oxidative stress and inflammation and by promoting cell migration.

### 3.7 PAL inhibits LPS-induced apoptosis by stabilizing mitochondrial membrane potential and regulating the apoptotic pathway

To clarify how PAL affects cell death after LPS exposure, we assessed mitochondrial membrane potential and apoptosis-related proteins using 5,5′,6,6′-tetrachloro-1,1′,3,3′-tetraethylbenzimidazolylcarbocyanine iodide (JC-1) staining and Western blot analysis. Control cells showed strong red fluorescence and little green fluorescence, indicating stable mitochondrial membrane potential. LPS-treated cells showed increased green fluorescence and reduced red fluorescence, consistent with mitochondrial depolarization and a greater tendency toward apoptosis. PAL treatment increased red fluorescence and reduced green fluorescence, with a more pronounced effect in the high-dose group than in the low-dose group (**P < 0.01; Figure 7A and B). Western blot analysis showed that LPS increased Bax and cleaved Caspase-3 and reduced Bcl-2. PAL reversed these changes, and the effect was more pronounced at the higher concentration. These findings indicate that PAL inhibits apoptosis by preserving mitochondrial membrane potential, restoring the Bcl-2/Bax balance, and suppressing Caspase-3 activation.

**Figure 7:**
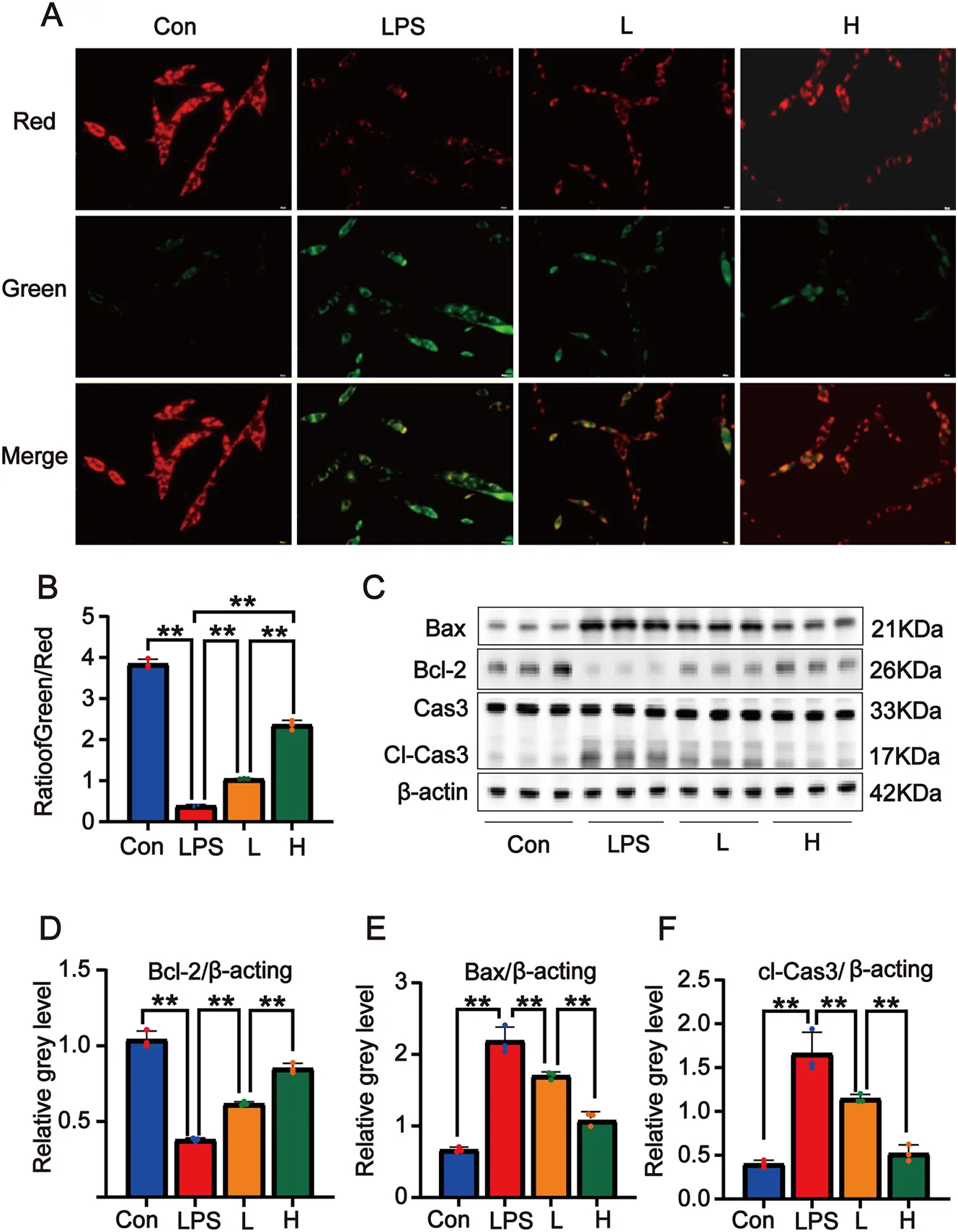
*In vitro* effects of PAL on mitochondrial membrane potential and apoptosis-related protein expression (A) JC-1 staining was used to assess changes in mitochondrial membrane potential. Red fluorescence represents J-aggregates and a normal membrane potential, whereas green fluorescence represents J-monomers and a reduced membrane potential. The merged image combines both signals. (B) Quantification of the red-to-green fluorescence intensity ratio (*n* ≥ 3). (C–F) western blot analysis of Bax, Bcl-2, and cleaved Caspase-3 in the mitochondrial apoptotic pathway, together with the corresponding quantification. *n* ≥ 3; *P < 0.05, **P < 0.01. L, LPS + 5 μg/mL PAL; H, LPS + 10 μg/mL PAL.

## 4 Discussion

### 4.1 Network pharmacology identifies potential PAL targets in LL-IRI

LL-IRI is a common pathological condition in vascular and trauma surgery. Its central mechanism begins with a post-reperfusion surge in reactive oxygen species (ROS), followed by oxidative stress, uncontrolled inflammation, and cell death, which ultimately lead to skeletal muscle injury, multiple-organ dysfunction, and poor clinical outcomes [24]. Although CR and CRRT can partially remove inflammatory mediators, their invasiveness, cost, and complication risk limit widespread use. Effective and noninvasive natural therapeutic strategies are therefore needed [25], 26]. PAL, a traditional Chinese medicine preparation, contains active constituents such as AU and has anti-inflammatory, antioxidant, and anti-apoptotic properties [27], 28]. This study provides a multidimensional assessment of the protective effects of PAL against LL-IRI and explores its underlying mechanisms.

We used network pharmacology to analyze the potential mechanism of PAL in LL-IRI. Active components and targets were identified from the TCMSP and BATMAN-TCM databases and intersected with LL-IRI-related targets from GeneCards and OMIM, yielding 145 shared targets. Component-target-disease and PPI networks highlighted TNF, IL-6, IL-1β, Bcl-2, Bax, and Caspase-3 as key targets involved in the pathological processes of LL-IRI. GO and KEGG enrichment analyses linked these targets to inflammation, oxidative stress, apoptosis, and the PI3K-Akt, MAPK, and VEGF pathways. These findings are consistent with studies of Huangjing and Jiegeng Gancao Tang and support the multi-component, multi-target, and multi-pathway characteristics of TCM [22], 23]. Molecular docking further showed that 10 key components, including MOL000449 and MOL000433, interacted with 10 core targets, including AKT1, Bcl-2, IL-1β, and TNF, with binding free energies of ≤−5.0 kcal/mol and, in some cases, <−7.5 kcal/mol. These results provide a structural basis for the *in vivo* and *in vitro* experiments and support the proposed multi-target mechanism of PAL.

Molecular docking is useful for evaluating interactions between active compounds and their targets. In this study, 10 major PAL components, including MOL000449 and MOL000433, were docked with 10 important targets selected on the basis of network and docking scores. All component-target pairs showed binding free energies of ≤−5.0 kcal/mol, and some were <−7.5 kcal/mol, indicating favorable binding. The docking models showed that PAL components interacted with key structural domains of AKT1, Bcl-2, IL-1β, and TNF through hydrogen bonds and other forces. These findings are consistent with previous reports that AU inhibits the NF-κB pathway by interacting with the HMGB1/TLR-4 complex [20]. and that plantamajoside interacts with key molecules in the ILK/c-Src/Akt pathway [18]. Together, these data provide a structural rationale for the multi-component and multi-target effects of PAL in LL-IRI.

### 4.2 In vivo confirmation of the combined protective effects of PAL against inflammation, oxidative stress, and cell death in LL-IRI

LL-IRI model rats developed lower-limb swelling, disorganized muscle fibers, inflammatory cell infiltration, and mitochondrial injury characterized by swelling and indistinct cristae [29]. These changes were consistent with previous reports and confirmed successful model establishment [30], 31]. After PAL treatment, the above pathological changes were markedly improved: lower-limb swelling was reduced, muscle fiber integrity was better preserved, inflammatory infiltration decreased, and mitochondrial morphology was largely restored. These findings indicate that PAL protects skeletal muscle from LL-IRI. Because mitochondria are a major source of ROS, mitochondrial structural injury is closely linked to oxidative stress and cell death. The apparent restoration of mitochondrial cristae after PAL treatment provides morphological evidence that PAL may preserve mitochondrial function and alleviate oxidative stress.

Apoptosis is a major mechanism of skeletal muscle cell death in LL-IRI and is regulated by the Bcl-2, Bax, and Caspase protein families [32], 33]. Multiplex immunofluorescence and TUNEL staining showed that LL-IRI increased Bax and cleaved Caspase-3 expression, reduced Bcl-2 expression, and markedly increased the number of apoptotic cells, indicating activation of the mitochondrial apoptotic pathway. PAL treatment reduced Bax and cleaved Caspase-3, restored Bcl-2, and decreased apoptosis, suggesting that PAL alleviates LL-IRI-induced skeletal muscle apoptosis by rebalancing apoptosis-related proteins [33]. These results are consistent with the findings of Du et al., who reported that plantamajoside attenuated hypoxia-reoxygenation injury in H9c2 cardiomyocytes by regulating the Bcl-2/Bax ratio and Caspase-3 activation.

### 4.3 *In vitro* confirmation of the mechanism by which PAL promotes muscle recovery and alleviates skeletal muscle cell injury

In LL-IRI, oxidative stress, inflammation, and apoptosis are initiated largely by the ROS surge that occurs during reperfusion. ROS can directly damage membrane lipids and trigger lipid peroxidatio [34]. Increased MDA levels in serum and tissue are commonly used as indicators of oxidative injury [35], 36] IL-1β is an important pro-inflammatory cytokine released after cleavage and maturation by the NOD-like receptor family pyrin domain containing 3 (NLRP3) inflammasome. It can amplify inflammation and activate apoptosis-associated kinases, thereby contributing to the extrinsic apoptotic pathway [37]. SOD converts superoxide anions into hydrogen peroxide, which is subsequently decomposed into water and oxygen by CAT [38]. In LL-IRI, reduced SOD and CAT activities impair ROS clearance and sustain a cycle of ROS accumulation, lipid peroxidation, and inflammatory activation. In the present study, PAL reduced MDA and IL-1β levels and restored SOD and CAT activities in the injury model. The concentration dependence of these effects suggests that PAL improves the pathological microenvironment by strengthening antioxidant defenses and suppressing inflammation. Western blot results further confirmed that PAL inhibited injury-induced activation of the apoptotic pathway. These coordinated effects may explain how PAL establishes a protective network involving antioxidant, anti-inflammatory, and anti-apoptotic actions.

Restoration of cell proliferation and migration is essential for tissue repair. In this study, an IC50 assay showed that 100 μg/mL LPS reduced C2C12 cell viability by 50 % after 48 h, and this condition was used to establish a skeletal muscle injury model. CCK-8 results indicated that PAL was not markedly cytotoxic at concentrations of 25 μg/mL or lower. PAL promoted C2C12 proliferation at 4–10 μg/mL and maintained a relatively high proliferation rate at 10–25 μg/mL; proliferation declined at concentrations above 25 μg/mL. Based on these results, 5 and 10 μg/mL were selected as low- and high-dose concentrations for subsequent experiments. The scratch assay showed that the high-dose PAL group had a greater wound closure rate than both the low-dose group and the control group, suggesting that higher PAL concentrations within the tested range more effectively promote the proliferation and migration of injured skeletal muscle cells. These findings provide a preliminary concentration range for future studies and indicate that PAL may not only limit injury but also support tissue recovery.

Mitochondrial membrane potential is essential for normal mitochondrial function and the prevention of apoptosis. JC-1 staining of LPS-treated C2C12 cells showed increased green fluorescence and reduced red fluorescence, indicating a loss of mitochondrial membrane potential and a tendency toward apoptosis. This observation is consistent with the finding that mPTP opening causes membrane potential collapse in tourniquet-induced hindlimb ischemia-reperfusion injury [4] and with reports of reduced mitochondrial membrane potential in models of neuronal oxidative stress [39]. PAL reversed these fluorescence changes in a concentration-dependent manner, suggesting that it stabilizes mitochondrial membrane potential and prevents apoptosis. Western blot analysis showed that PAL reduced Bax and cleaved Caspase-3 and increased Bcl-2, consistent with the *in vivo* results. These effects suggest that PAL interferes with the mitochondrial apoptotic pathway by restoring the Bcl-2/Bax balance and limiting Caspase-3 activation, in agreement with previous findings on plantamajoside and AU [18], 20].

## 5 Conclusions

Using a multidimensional experimental framework, this study examined the protective effects and underlying mechanisms of PAL in LL-IRI. Neither the animal experiments nor the cell experiments included a positive control group (e.g., N-acetylcysteine, NAC), making it impossible to directly compare the efficacy of PAL with first-line clinical antioxidant/anti-inflammatory agents in a head-to-head manner, which to some extent limited a thorough evaluation of the clinical translational significance of this study. Furthermore, combination regimens of PAL with existing clinical drugs (e.g., NAC) were not explored, and the synergistic or antagonistic effects of such combination therapy require further validation. The results indicate that PAL constituents act cooperatively on three major pathological processes: oxidative stress, inflammation, and apoptosis, with mitochondrial stability serving as a central mediator. PAL may reduce early oxidative injury by limiting ROS-associated damage and enhancing endogenous antioxidant defenses, attenuate inflammation by suppressing pro-inflammatory cytokines such as IL-1β, and inhibit mitochondrial apoptosis by preserving membrane potential and regulating the Bcl-2/Bax ratio. These findings provide a scientific basis for further investigation of PAL as a candidate for preventing or treating LL-IRI and illustrate the value of combining network pharmacology with experimental validation in the modernization of traditional Chinese medicine.

This study provides preliminary evidence that PAL protects against LL-IRI, while also identifying questions that must be addressed before translation. Long-term efficacy and safety were not comprehensively assessed, particularly with respect to sustained functional recovery in animal models and pharmacotoxicological evaluation. For example, its main active component aucubin is metabolized extremely rapidly in the intestinal tract; how to improve its oral bioavailability also requires further investigation in the future. The pharmacodynamic basis and metabolic pathways underlying the effects of PAL also remain unclear. In addition, the principal active components and optimal combinations have not yet been defined, which limits standardization and clinical translation. Mechanistic analyses focused mainly on mitochondrial function and apoptosis and did not examine upstream signaling pathways or other forms of regulated cell death. Recent evidence suggests that PANoptosis contributes to ischemic injury in the heart, brain, and kidney [40], but whether it is involved in LL-IRI remains unknown. Future research should include positive control groups such as NAC, systematically evaluate the efficacy and safety of PAL alone and in combination with clinical drugs, screen and prepare high-purity active monomers, deepen the investigation of signaling pathway mechanisms using multi-omics technologies, and establish long-term protocols for evaluating long-term effects and safety. The roles of PANoptosis and the PANoptosome in LL-IRI should also be investigated, together with potential combinations of key PAL constituents and existing clinical drugs, to support clinical translation.
